# The effect of deferoxamine injection on composite graft survival in rabbits

**DOI:** 10.1016/j.amsu.2020.11.029

**Published:** 2020-11-21

**Authors:** Rianto Noviady Ramli, Agus Santoso Budi, Sitti Rizaliyana, Aditya Rifqi Fauzi

**Affiliations:** a Plastic Reconstructive, and Aesthetic Surgery Division, Department of Surgery, Faculty of Medicine, Public Health and Nursing, Universitas Gadjah Mada/Dr. Sardjito Hospital, Yogyakarta, 55281, Indonesia; b Department of Plastic Reconstructive, and Aesthetic Surgery, Faculty of Medicine, Universitas Airlangga/Dr. Soetomo Hospital, Surabaya, Indonesia

**Keywords:** Composite graft, Survival rate, Deferoxamine, PRP

## Abstract

**Background:**

Composite graft as a reconstructive therapy option has limitations in size so that it is easily necrotic. Deferoxamine administration has been associated with increased neo-vascularity in wounds. We aimed to compare the administration of deferoxamine and Platelet-Rich Plasma (PRP) injection in a composite graft in rabbits.

**Methods:**

Thirty New Zealand rabbits were divided into three groups; the control group, the deferoxamine group, and the PRP group. The composite graft with a diameter of 2 cm was taken and replanted after rotating it 180°. The mean graft viability and the mean number of capillaries were evaluated on day 7 (POD 7) by macroscopic and histological evaluation using Hematoxylin-Eosin staining.

**Results:**

While the mean number of capillaries was not significantly different in control, deferoxamine, and PRP groups (*p* = 0.21), the mean survival rate in the control, deferoxamine, and PRP groups reached a significant level with *p*-value of 0.006 (66.6% *vs.* 63.8% *vs.* 99.6%, respectively).

**Conclusions:**

Deferoxamine group had the highest number of capillaries, but had the lowest survival rate. In the PRP group, it had the lowest number of capillaries, but had the highest survival rate.

## Highlights

• Composite grafts yield better results than skin grafts in plastic and reconstructive surgery.
• The failure rate of composite graft is quite high due to limitations of re-vasculature.
• The usage of deferoxamine might have a beneficial effect on increasing the graft survival.

## 1 Background

Reconstruction of physical defects or deformities due to several conditions, either congenital or acquired back into normal form and function, is the main goal of plastic and reconstructive surgery. Composite grafts yield better results than skin grafts in discrepancies in color and texture at the donor site, causing less scar contracture, because more tissue structure is provided by transferring 2 or more different tissue types. However, the composite graft has limited graft viability when the recipient defect is more than 1.5 cm in diameter [1].

With the limitation of re-vasculature, the failure rate of composite graft is quite high [[2], [3], [4]]. Angiogenesis which plays a role in graft re-vascularization can be increased by direct injection of growth hormones such as fibroblast growth factor (FGF), platelet derived growth factor (PDGF) and vascular endothelial growth factor (VEGF). The important role of VEGF in angiogenesis is as a specific mitogen factor for endothelial cells, which stimulates the formation of new blood vessels and increases their permeability. In PRP, there are many of the growth factors mentioned above which are proven to increase the viability of the composite graft [5].

However, the availability of PRP in local health services is an obstacle. Therefore, the use of alternative drugs needs to be considered. Deferoxamine as an iron chelating agent has been shown to have a wound healing effect [6,7]. We aimed to compare the administration of deferoxamine and Platelet-Rich Plasma (PRP) injection in a composite graft in rabbits.

## 2 Material and methods

### 2.1 Animal models

Thirty male New Zealand rabbits, aged 9–12 months, weighing 2500–3000 g were isolated under standard conditions for 7 days. Rabbits that were sick, had an infection during the procedures, or died during the procedures were excluded from this study. The work has been reported in line with the ARRIVE guideline [8].

### 2.2 Experimental procedures

**Fig. 1:**
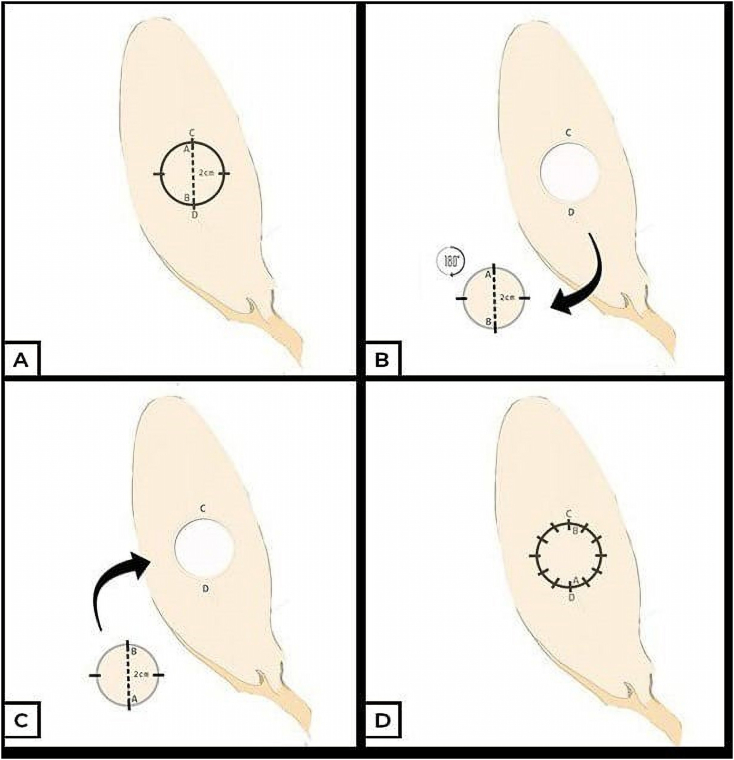
Composite graft treatment scheme.

All experiments were performed in the Faculty of Veterinary Medicine of Universitas Airlangga. These experimental procedures were performed with the prior approval of the Medical and Health Research Ethics Committee of the Faculty of Medicine, Universitas Airlangga (2. KE.131.07.2018). Each rabbit was injured in the ear with a diameter of 2 cm, full thickness through and through. Then the composite graft on the rabbit ears consisting of skin tissue, sub cutis, and rabbit auricula cartilage in the form of a circle 1 cm in diameter was excised from the rabbit auricula, rotated 180°, and sewn back (Fig. 1).

In group I, no subcutaneous injection was given. Group II was given subcutaneous 100 mg/kg deferoxamine injection (Novartis Pharma Stein AG Stein, Switzerland), in all four quadrants outside the composite graft using a 1 cc injection syringe one day before the composite graft, post graft treatment procedure, and within the first 3 days. Group III was given PRP injection of 0.5 ml subcutaneous, post graft, and within the first 3 days. The viability of the graft was checked every day and the 7th day was assessed with Visitrak (Kyros International Inc., USA). On the 7th day, the skin was harvested for examination of vascular density.

**Fig. 2:**
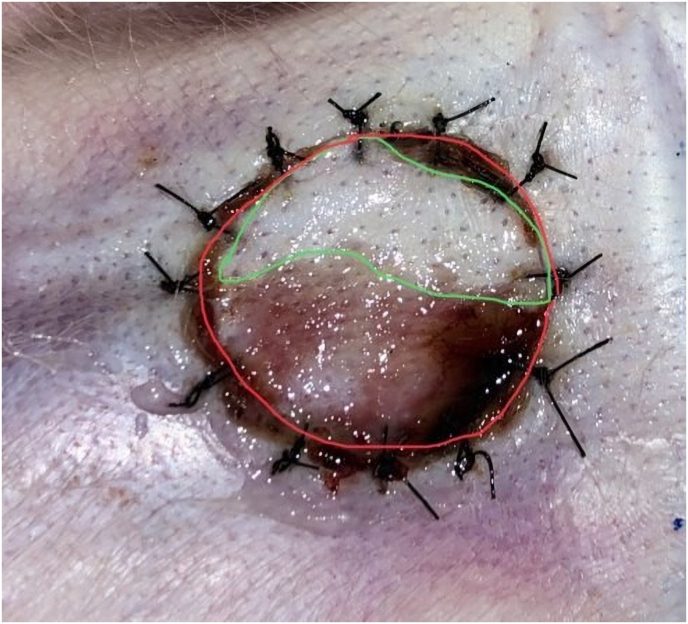
Composite graft treatment viability.

Composite graft was assessed for its viability based on macroscopic observations, the non-viable tissue was described by the presence of necrotic, darker color accompanied by dry tissue, such as tanning in the red marker areas in Fig. 2, while the viable graft fingers were not visible as green areas.

### 2.3 Statistical analysis

Kruskal-Wallis and Mann-Whitney test were used to evaluate the graft survival between groups. Independent *t*-test was used to evaluate the capillary count between groups. For statistical analyses, a *p*-value of less than 0.05 was considered to be significant and 95% confidence interval was used in this study.

## 3 Results

The graft survival of rabbits between control *vs.* deferoxamine *vs.* PRP reached a significant level (66.6% *vs.* 63.8% *vs.* 99.6%; *p* = 0.006). Further analysis showed that control *vs.* PRP, and deferoxamine *vs.* PRP differ significantly with *p*-value of 0.003 and 0.004, respectively (Table 1).

**Table 1:** Graft survival analysis.

	Treatment group	N	Mean rank	p
Graft survival	Control	8	10.75	0.006*
	Deferoxamine	10	11.25	
	PRP	10	20.75	

Graft survival	Control	8	9.38	0.928
	Deferoxamine	10	9.60	

Graft survival	Control	8	5.88	0.003**
	PRP	10	12.4	

Graft survival	Deferoxamine	10	7.15	0.004**
	PRP	10	13.85	

PRP: Platelet-rich plasma, *p < 0.05 is considered significant by Kruskal-Wallis, **significant by Mann-Whitney.

However, in terms of capillary counts, no significant difference was found between control *vs.* deferoxamine (204.7 ± 44.4 *vs.* 212.8 ± 98.2, *p* = 0.82), and deferoxamine *vs.* PRP (212.8 ± 98.2 *vs.* 160.8 ± 39.9, *p* = 0.147). But, in contrast, control *vs.* PRP showed a significant difference (204.7 ± 44.4 *vs.* 160.8 ± 39.9, *p* = 0.042) (Table 2).

**Table 2:** Capillary count analysis.

	Treatment group	N	Mean ± S.D	p
Graft survival	Control	8	204.7 ± 44.4	0.209
	Deferoxamine	10	212.8 ± 98.2	
	PRP	10	160.8 ± 39.9	

Graft survival	Control	8	204.7 ± 44.4	0.82
	Deferoxamine	10	212.8 ± 98.2	

Graft survival	Control	8	204.7 ± 44.4	0.042*
	PRP	10	160.8 ± 39.9	

Graft survival	Deferoxamine	10	212.8 ± 98.2	0.147
	PRP	10	160.8 ± 39.9	

PRP: Platelet-rich plasma, S.D: standard deviation, *p < 0.05 is considered significant using independent *t*-test.

## 4 Discussion

Deferoxamine group has the lowest graft viability rate with only 63.8%, this is in contrary to the results obtained by Wang's [9] study, namely the injection of deferoxamine in mouse back skin flaps, which was started 1 day before and continued for 3 days after flap elevation is 90.1% at a dose of 100 mg/kg.

The viability of a composite graft depends on several factors, such as skin grafts. The viability of the composite graft comes from serum inhibition, re-anastomosis of blood vessels, and neovascularization [10]. However, unlike skin grafts, composite grafts have a layer of cartilage, which can act as a mechanical barrier that limits the vascularization of the wound bed. Thus, it is conceivable that revascularization via the dermis connection to the dermis at the wound edge is more important for the survival of the composite graft, and in turn limits the size of the composite graft [11]. Because it is like a skin graft, the viability of the composite graft is influenced by good recipient vascularity, accurate contact between the graft and recipient, and immobilization [12].

Several conditions that can reduce the viability of the composite graft, such as the pressure on the composite graft can damage the recanalization and neovascularization process, this is because the pressure makes the ischemic tissue, ischemic tissue releases inflammatory mediators that cause edema, moreover will cause vascular thrombosis [10]. On the basis of this, the low viability of the composite graft in the deferoxamine group can occur due to the large number of substances injected into the ears of rabbits, it has a body weight of 2 kg, based on the dose of 100 mg/kg each ear and the deferoxamine content of 100 mg/cc, so each ear gets 2 cc, or each quadrant received 0.5 cc of deferoxamine. During this treatment, 0.5 cc which is injected into the subcutaneous can create bulging and edema in the recipient subcutaneous layer, which will interfere with the vascular inosculation process from the recipient wound edge to the wound edge of the composite graft, so that the process has an effect on the revascularization process that is not working well and the end result will affect the viability of the composite graft. This mechanism was repeated four times according to the schedule of administration in the deferoxamine treatment group up to day 7.

The high viability results were in the PRP group, which had a mean graft viability of 99.6%. This is in line with Choi's [5] study conducted on composite rabbit ear grafts, namely that PRP administration to the recipient area 3 days before grafting had a graft survival rate of 97%, compared to administration immediately after graft (69.2%), 3 days after graft (55.7%) and control (40.7%).

Platelet-rich plasma (PRP) contains various growth factors such as platelet derived growth factor (PDGF), transforming growth factor - β (TGF-β), vascular endothelial growth factor (VEGF), endothelial growth factor (EGF), insulin-like growth factor (IGF1), endothelial cell growth factor (ECGF) [13]. The growth factors released by PRP can increase epithelialization, the amount of collagen, wound strength, epidermal regeneration, stimulate angiogenesis, accelerate homeostasis, therefore the use of PRP can increase skin flap viability [[14], [15], [16]]. The use of PRP can increase tissue regeneration and reduce the risk of infection, pain, and blood loss. In addition, PRP may suppress the release of cytokines, suppress inflammation, and will increase tissue regeneration. Angiogenesis is known to take 3–5 days [10].

Despite having a low mean graft viability in the deferoxamine treatment group, the histological findings in the form of capillary count were the highest of the other groups, although statistically there was no significant difference. Thangarajah [17] states that deferoxamine makes HIF-1α stable in wounds, which in turn stimulates the expression of VEGF which ultimately increases neo-vascularization. Vascular endothelial growth factor (VEGF) is the permeability factor of blood vessels released from the wound epithelium and extracellular matrix by proteases from endothelial cells, stimulating endothelial cell proliferation and increasing vascular permeability. This affects plasma protein extravasation and creates a temporary support structure through which activated endothelial cells, leucocytes and epithelial cells can further migrate [18]. Therefore, the number of capillaries was more in the deferoxamine group.

The results differed in the PRP group, the number of capillaries in the PRP group was the least, this could be due to the wound healing process running very well. Li [19] found a significant increase in PDGF expression after 8 h, and lasted from 3 to 7 days. The release of PDGF into the skin can have a chemotactic effect on monocytes, neutrophils, fibroblasts, mesenchymal stem cells. Platelet derived growth factor (PDGF) is also a strong mitogen for fibroblasts and smooth muscle cells and is involved in the wound healing phase (i.e., angiogenesis, fibrous tissue formation, and re-epithelialization) [15]. The histologic findings of the PRP group appear that granulation tissue is reduced and vascular tissue is also reduced, which is a sign that the tissue is healing.

Our study is not without limitation. First, we did not measure level of the growth factor to see the effect of injection of deferoxamine and PRP on the composite graft. Second, we did not perform histological examination on the 3rd day after the graft as a comparison to prove that the PRP administration also increase the number of capillaries before experiencing a decrease in the number on the 7th day. Therefore, further study with larger sample is necessary to confirm our findings.

## 5 Conclusions

Administration of deferoxamine three days before grafting can significantly increase the viability of composite graft. Our study implies that the usage of deferoxamine might have beneficial effect on composite graft viability.

## Provenance and peer review

Not commissioned, externally peer-reviewed.

## Ethical approval

Not applicable.

## Sources of funding

The authors declare that this study had no funding source.

## Author contribution

Rianto Noviady Ramli conceived the study and approved the final draft. Aditya Rifqi Fauzi drafted the manuscript. Agus Santoso Budi and Sitti Rizaliyana critically revised the manuscript for important intellectual content. All authors read and approved the final draft. All authors facilitated all project-related tasks.

## Trial registry number

Not applicable.

## Guarantor

Rianto Noviady Ramli.

## Consent

Not applicable.

## Declaration of competing interest

No potential conflict of interest relevant to this article was reported.

## Abbreviations

POD: Postoperative day

## Appendix A Supplementary data

Application Application
